# Understanding the holistic experiences of living with a kidney transplant: an interpretative phenomenological study (protocol)

**DOI:** 10.1186/s12882-020-01860-3

**Published:** 2020-06-11

**Authors:** C. McKeaveney, H. Noble, A. E. Courtney, P. Gill, S. Griffin, W. Johnston, A. P. Maxwell, F. Teasdale, J. Reid

**Affiliations:** 1 Queen’s University Belfast, Belfast, UK; 2 Belfast Health & Social Care Trust, Belfast, UK; 3 Cardiff University, Cardiff, UK; 4 University Hospital of Wales, Cardiff, UK; 5 Northern Ireland Kidney Patients Association, Belfast, UK; 6 Kidney Care UK, Alton, UK

**Keywords:** Qualitative, Kidney, Transplant, Holistic, Well-being, Interpretative phenomenological analysis

## Abstract

**Background:**

Currently very little is known about the perceptions and experiences of kidney transplant recipients from a qualitative perspective. As highlighted by the European Kidney Health Alliance recommendations, providing holistic care to kidney patients is important however this is currently an unmet care need in renal disease. It is imperative to understand patient experiences to ensure that they are included in key strategies and future renal service planning. Ignoring these important patient views means that there is a significant risk of inappropriate renal service provision and lack of adequate support impacting on overall health.

**Method:**

A purposive sampling strategy will recruit individuals currently living with a kidney transplant, 6 months to 5 years post-transplant. A maximum of 30 patients will be recruited between two Regional Nephrology units within the United Kingdom via clinical gatekeepers. In-depth interviews will be undertaken with participants living with a kidney transplant across the two sites. Interviews will be digitally-recorded, transcribed verbatim and subjected to interpretative phenomenological analysis.

**Discussion:**

Renal healthcare professionals need to understand more than the biological impact of receiving a kidney transplant. Understanding the holistic and multi-domain experiences that these patients experience will help healthcare professionals to recognize the needs of this group and ensure more responsive care.

## Background

Kidney transplantation will improve quality of life however this tends to be lower when compared to general population [1, 2]. Many patients aim to restore a sense of ‘normality’ to their lives with a functioning kidney transplant [3]. However, a number of factors may impact on this leading to significant physical and psychosocial challenges. Kidney transplant recipents can experience significant medication side effects and develop comorbidities that reduce psychosocial wellbeing and quality of life [4]. Kidney transplant recipients can also feel a tremendous sense of gratitude at receiving the ‘gift of life’ however the tyranny of the gift causes patients to experience significant negative symptoms and emotions including fear of rejection, post-transplant complications, existential uncertainty and guilt [5]. Stressors can also include changes in family dynamics, dietary constraints, time restrictions, functional limitations, medication side effects, changes in employment, relationships with staff, change in sexual function, cognitive dysfunction and awareness of impending death [6–8].

There are quantitative data describing how persons recover following transplant surgery e.g. number of hospitalisations, renal function, medication use at various time points. Nevertheless, it is unclear how persons with a kidney transplant learn to adapt to life post-transplant. The dearth of such qualitative information following transplantation represents a gap in the current literature [9]. The research team includes patient representatives as well as renal consultants and three experienced qualitative academics. This proposal has culminated from engagement from this group and reflects recent clinical guidelines by the Renal Association and British Transplantation Society emphasizing holistic care for transplant recipients [10]. High patient and graft survival rates after kidney transplantation have led to a growing population of individuals living with kidney transplants. Unfortunately, the provision of psychological and social support for patients after they have received a kidney transplant is well recognized to be inadequate [11]. Quantitative data has also shown that these patients can experience a diminished quality of life and increased risks of depression, anxiety and dissociative disorders [5]. To date, there is limited in-depth qualitative data to understand these experiences. Thus, there is an urgent need for research that seeks to understand the important aspects of living with a kidney transplant. The aim of this study is to provide an in-depth exploration of life after kidney transplantation to identify patient needs within nephrology and build an evidence base to support increasing provision in relation to supportive services, such as support groups, psychological therapy and educational programs [12].

The proposed exploratory research will use an interpretative phenomenological approach to illuminate the lived experiences of patients. Interpretative phenomenology is designed to gain insight into the participant’s world and create a deeper understanding of the quality and texture of the experiences of the phenomena, thus it is appropriate to address the research aim of this study [13]. This research approach aims to reveal the depth of individual experiences by exploring text and verbatim transcripts from interviews conducted with participants. Thus, this philosophical approach will be adopted within this study. Furthermore, it is the only qualitative research approach that takes the subjective experiences of the research participants as its focus [14]. This will enable the personal meaning of living post-transplant for each of the research participants to be elucidated.

## Methods

### Aim

The aim of this study is to explore the lived experience of having a kidney transplant.

### Design

This study will include a qualitative methodology underpinned by Heideggerian phenomenology which focuses on interpreting and understanding the meaning of lived experiences [15, 16].

### Setting

The study will be conducted at two Regional Nephrology Units within the United Kingdom.

### Study participants

A purposive sample of 30 patients will be recruited across two regional sites (15 patients per site; purposive recruitment to ensure a range of perspectives are included). Inclusion and exclusion criteria are outlined in Table 1.

**Table 1:** Eligibility criteria

Inclusion	Exclusion
18 years of age and over	Under 18 years of age
Received first kidney transplant	Has had a previous kidney transplant
Between 6 months and 5 years post-transplant	Transplant less than 6 months or more than 5 years ago
English speaking	Non-English speaking
Ability to provide informed consent	Cognitive impairment (as assessed by their consultant or health care team)
Deceased or living donor; prior dialysis or pre-emptive transplant	

Sampling will be driven by the aspiration of learning in detail about the depth of experience of the research participants. Thus, the final sample size will be determined by the research team when it is agreed data saturation has been achieved [17]. Clinical gatekeepers will help to identify patients and provide an online study link to the invitation letter, patient information sheet, consent form which includes a separate request for telephone and email details. Upon receipt of contact information, the researcher will make a follow-up telephone call to discuss participation and answer any questions potential participants may have. If the participant is still willing to take part, a convenient time will be arranged for the interviews to be conducted. Data will be collected via semi-structured interviews (one per participant), the appropriateness of this data collection technique in phenomenological research is outlined in the literature [18]. Participants will be offered an online (e.g. Skype) or a telephone interview (as per Health Research Authority [19] COVID-19 guidelines). Previous research has demonstrated that when using semi-structured interviews to explore sensitive issues, depth of data is not lost in using alternative methods of data collection such as the telephone [20].

### Study materials

In line with phenomenological research, a broad interview guide for use within the semi-structured interviews, has been derived from the literature and from iterative feedback from the research team. Semi-structured interviews will use an inductive format and utilize non-directive, open ended questions to aid communication. A semi-structured interview guide will be used flexibly within each interview, the appropriateness of which is outlined within the literature [18, 21]. The broad domains will allow room to phrase questions spontaneously [22], to probe, clarify and reflect. This will facilitate exploration of emerging areas raised by the participants (see supplementary file 1). All interviews will be digitally recorded and transcribed verbatim for data analysis. This will be entered on a data management tool, NVivo 12 [23]. Interpretative Phenomenological Analysis (IPA), which is a method of analysis appropriate for exploring in detail participants’ perceptions of the phenomenon under study, will be used within this research.

### Analysis

The term IPA is used to denote the dual nature of this analytic approach, which draws on phenomenology [24] and is connected to hermeneutics, which focuses on achieving a common understanding of meaning [18]. IPA provides a narrative account which provides a greater understanding of such human experiences that can be used to inform health care policy and practice. More importantly, IPA can potentially enrich an area previously only studied through quantitative measures. Therefore, the analysis presented in this study endeavours to display the diversity of viewpoints among the participants. The process of IPA as outlined by Smith et al. [18] begins with listening to the first recorded interview and reading the interview transcript several times. Statements that reveal the lived experience of living with a transplant will be identified and organised into themes. Smith et al. [18] suggests on a separate sheet, emerging themes should be listed and any connections between them established. Also ensuring any connections are accurate by checking back upon the initial transcripts. The next stage is to produce a table of themes. Then analysis will continue with the remainder of the cases. The themes that emerge from the first transcript will be used as an outline for subsequent interview analysis. All new themes in subsequent transcripts will be clarified with the previous and will continue back and forth between the cases in this way. Finally, by clustering themes together, a master list of themes will be produced. Two members of the team (JR and CM) will independently code data and then compare coding to help refine the final themes from analysis.

### Ethical considerations

Confidentiality and data protection procedures will be applied as minimum standards. These include voice files/transcripts being numbered rather than documenting the participants’ names, the use of pseudonyms in transcripts/quotations, access to raw data being confined to the research team, secure storage of voice files and transcripts, voice files deleted after transcription. The research team are experienced in qualitative research and as such are cognisant of the fact that qualitative interviewing has the potential to induce or exacerbate emotional distress, therefore a distress protocol (adapted from Draucker et al. [25]) will be adhered to. Informed consent from participants will be obtained prior to interview.

### Participant and public involvement (PPI)

Members of the research team are also members of the Northern Ireland Kidney Patients Association (NIKPA) and Kidney Care UK (KCUK) (WJ, FT). Members have participated in the design, protocol development and patient facing materials. Collaboration will continue with dissemination of findings.

## Discussion

It is clear psychological distress experienced through illness or life-sustaining treatment is not eliminated as a result of receiving a transplant. Not only do kidney transplant recipients continue to live with a significant chronic illness, but they also carry substantial physical and emotional stressors originating from the progression and irreversible stage of their renal disease [26]. Research has clearly demonstrated that robust psychological and social support is associated with better survival, medication adherence, reduced psychological distress, and improvements in health-related quality of life (HRQoL) including social functioning [27]. Therefore, individuals with chronic kidney disease require holistic care that includes a range of therapies encompassing social, psychological and pharmacological support [28].

While quantitative evidence has demonstrated significant quality of life and well-being concerns in these patients after transplantation, it is important to further understand these experiences to provide appropriate support for this unique patient group. Kidney transplant teams must ensure optimal clinical outcomes and psychosocial outcomes; however, the latter continues to be an unmet care need in renal disease. The provision of psychosocial care is limited withn the United Kingdom as kidney transplant recipients are less likely to access appropriate holistic care, including psychological and social support [11]. There is a need to provide a patient-led and patient-centred evidence base. This will provide an understanding of the holistic perspective and experiences of transplant patients. This will also aid greater awareness, ensure clear recommendations are provided and appropriate psychosocial support and interventions are available within routine practice.

## Conclusions

The proposed research will provide in-depth understanding into a highly complex phenomenon. Little is known about the holistic impact on kidney transplant recipients and this research will provide novel insights for future research and will add to the ongoing development and improvement of psychosocial services within nephrology. With a better understanding of the trajectory of the transplant treatment journey there is potential for new and improved information, advice and support at all stages of the treatment journey including pre-dialysis, dialysis and post-transplant stages.

## Supplementary information

**Additional file 1.** Interview topic guide.

## Data Availability

The datasets generated and analysed during the current study will be made available in the QUB repository (http://pure.qub.ac.uk/portal/en/ datasets/search.html).

## Abbreviations

ESKD: End-stage kidney disease
NIKPA: Northern Ireland Kidney Patients Association
KCUK: Kidney Care UK
REC: Research Ethics Committees
HRQoL: Health-related quality of life
PPI: Patient and Public Involvement
NICE: National Institute for Health and Care Excellence
UK: United Kingdom
HCP: Healthcare professional
IPA: Interpretative Phenomenological Analysis
