# Supplementary material for: Understanding the holistic experiences of living with a kidney transplant: an interpretative phenomenological study (protocol)
Source: BMC Nephrol. 2020 Jun 11;21:222. doi: 10.1186/s12882-020-01860-3 (PMC7289222; doi:10.1186/s12882-020-01860-3)
Supplement: Supplementary file 1 — Additional file 1. Interview topic guide. [file 12882_2020_1860_MOESM1_ESM.docx]

Additional file 1. Topic guide

| Main topics | Prompts |
| --- | --- |
| 'Open invitation statement' | Tell me a little about your transplant treatment journey? Prompts relating to ACE (adverse childhood experiences), age of onset of kidney disease, experience of dialysis (e.g. duration) or pre-emptive work-up. |
| Emotional | Can you tell me what feelings you experience day to day living with a kidney transplant? Prompts relating to emotional impact been of living with a kidney transplant. |
| Social | Can you tell me about your social life since your transplant? Prompts related to relationships with your children/partner/close friends/relatives. |
| Physical | What has been the impact of your transplant on you physically? Prompts relating to activities of daily living, independence. |
| Financial | Can you tell me about the financial practicalities you experience living with a kidney transplant? Prompts relating to childcare, housing, insurance, financial, transport, work. Could you tell me about any supports and/or barriers you have faced relating to these aspects of living with a kidney transplant e.g. financial, employment, and education? |
| Support | In thinking about adapting to life after your transplant, can you describe to me what coping skills you have used? What kind of support would you like as a kidney transplant patient? Prompts relating to type, medium, location, of potential supports. |
